# North American Birds and West Nile Virus

**DOI:** 10.3201/eid1008.AC1008

**Published:** 2004-08

**Authors:** Polyxeni Potter

**Affiliations:** * Centers for Disease Control and Prevention, Atlanta, Georgia, USA

**Keywords:** Art and science, emerging infectious diseases, biologic agents, disease emergence, cover text, North American Birds, West Nile Virus, Edgar Allan Poe, Raven

**Figure:**
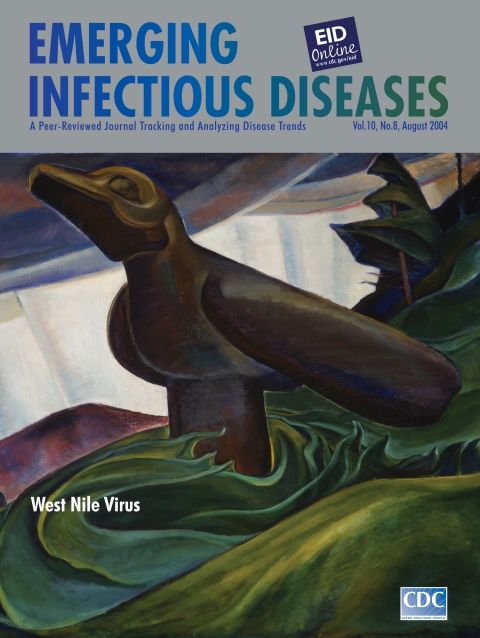
Emily Carr (1871–1945). Big Raven (1931). Oil on canvas, 87.3 cm x 114.4 cm, Vancouver Art Gallery, Emily Carr Trust

"Prophet!" said I, "thing of evil!—prophet still, if bird or devil!
Whether tempter sent, or whether tempest tossed thee here ashore,
Desolate, yet all undaunted, on this desert land enchanted—
On this home by horror haunted—tell me—tell me I implore!"
Is there—is there balm in Gilead?—tell me—tell me I implore!"

Edgar Allan Poe "The Raven"

"Not far from the house sat a great wooden raven mounted on a rather low pole; his wings were flattened to his sides.… His mate…had rotted away long ago, leaving him moss-grown, dilapidated and alone…these two great birds had been set, one on either side of the doorway of a big house that had been full of dead Indians who had died during a smallpox epidemic. Bursting growth…grew up round the…raven, sheltering him from the tearing winds now that he was old and rotting...." wrote Emily Carr in Klee Wyck, the best-selling book of short stories about her many visits to Native villages near Victoria, Canada, where she was born (*1*).

Artist, author, and passionate advocate of trees and birds, Carr drew inspiration and focus from decaying aboriginal artifacts that littered the wilderness of her beloved British Columbia. Many of her works seem haunted by these artifacts' legacy of epidemics and death. With a "smothering darkness," descended perhaps from her own Anglo-Victorian culture's fear of the primeval forest, she conveyed the frailty of human efforts against the power of the woods and the spirits in them (*2*).

Carr pursued an artistic career from age 16 and attended the California School of Design in San Francisco. She taught art; traveled to England, France, and the wilds of Canada's Pacific Coast in search of personal style; and exhibited widely, in spite of financial constraints and ill health from heart disease and frequent bouts of depression. Her dedication to the natural world and her belief in the mystical and spiritual connection between all things culminated during the latter part of her life (1933–1936) in landscapes of "exceptional spontaneity and expressiveness" (*2*).

The late 19th century witnessed sweeping cultural changes. Existing values were questioned in science, philosophy, and the arts, at the individual and social levels. This was the era of, among countless greats, Robert Koch, Louis Pasteur, Charles Darwin, Marie Curie, Albert Einstein, Fredrich Nietzsche, Sigmund Freud, George Eliot, Walt Whitman, Mary Cassatt. Artists were moving away from descriptive likeness toward visual impression of objects. During her travels to Europe, Carr explored modernism and pondered its "big ideas" in the context of the "big land" of her childhood, adopting new styles, transcending her own experience, creating potent landscapes for the world (*3*).

Like fellow North American artists Georgia O'Keeffe and Frida Kahlo, Carr turned for authenticity to nature and to her own and Native cultures. The wilderness of British Columbia and southern Alaska and the work of Pacific Coast communities roused her artistic imagination. She came to view nature as anthropomorphic and trees, stars, rocks, and all natural forms as symbolic reality with which she could identify—once, in a rare self-portrait, she painted herself in the form of a tree (*4*).

Carr continued to paint decaying tribal artifacts as she experimented with modern techniques. And by adapting the structuring influence of cubism to paintings of Pacific Coast tribal art, she did more than preserve this art from extinction. She brought history full circle by reviving and reformulating artifacts whose kin, the tribal art of Africa and South Pacific, had greatly influenced the development of cubism in France (*2*).

Big Raven evolved from the watercolor image of a Haida totem pole Carr had painted at Cumshewa, Queen Charlotte Islands, almost 20 years earlier. "I want to bring a great loneliness to this canvas and a haunting broodiness, quiet and powerful," the artist wrote in her journal (*4*). Broodiness notwithstanding, Big Raven is full of energy and movement. The sky and landscape are sculptured, as solid and heavy as the raven itself, yet their interlocking elements are spirited. They heave and swell, their scalloped edges undulating in a powerful swirl around the massive bird.

This remnant of a vibrant household struck down by the plague of its time stands a lonely symbol of passing plagues in Carr's green sea of anthropomorphic nature. Perched low, impassive, silent, and seemingly unmoved, it feigns obscurity and anonymity, but the upward avian thrust, grave countenance, and ghastly glare label it prophet of doom.

A single bird like Carr's lonely oracle sends proper warning. A population of birds in distress or dying is a far more useful sentinel; watchful tracking of their predicament informs the ecology and dispersal of disease. Animals turn sentinels as their deaths presage human illness on the epidemic curve. Dying prairie dogs signal human plague in the American Southwest (*5*). Horses dying of eastern equine encephalomyelitis point to increased spread of virus in a community (*6*). When an "Old World epizootic strain," West Nile virus, made its way across North America, from the Atlantic to the Pacific Coasts, from Canada into tropical regions and the Caribbean (*7*), unexplained avian deaths sometimes occurred weeks before human West Nile virus encephalitis cases. And dying birds of the crow family, including ravens, foretold human infection in the New World as they likely did in ancient Babylon (*8*).
